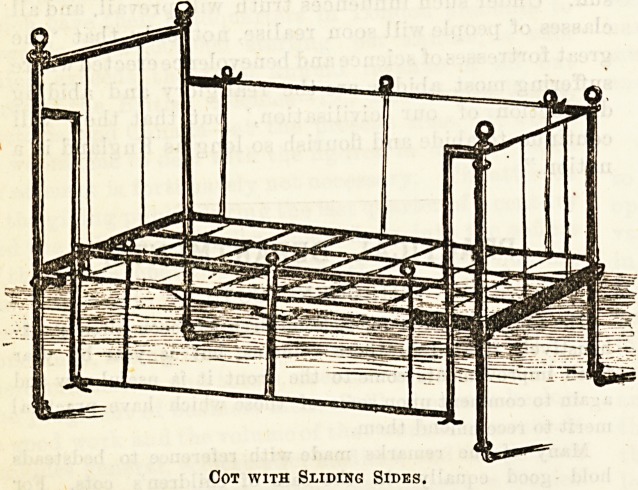# Children's Cots

**Published:** 1895-01-05

**Authors:** 


### PRACTICAL DEPARTMENTS.

### CHILDREN'S COTS.

The subject of cots for sick children has been already exhaustively treated in these columns, but as year by year fresh improvements come to the front it is useful now and again to comment upon some of those which have practical merit to recommend them.

Many of the remarks made with reference to bedsteads hold good equally in the case of children's cots. For institution use it is naturally of the first importance that they shall fulfil certain obvious conditions. They must be as simple in shape as is compatible with the necessary strength; they must be light, as well as strong in make; and they must be provided with good castors, to permit of being easily moved, without marking and scratching the floor. It is, of course, indispensable that the sides shall be easily removable, whilst it is also desirable that head and foot pieces should fulfil the same conditions. The old arrangement whereby the sides could only be removed by the lengthy process of unscrewing the corner knobs and bodily taking away the pieces has now been replaced by far more convenient methods.

COT WITH FALLING SIDES.

Our first illustration shows one of the usual drop-sided cots. The side pieces simply unhook and fall over, a feat accomplished in a moment, and perhaps somewhat too easily for a restless occupant. Such an arrangement is safe enough when the small invalid is unable to move about at his or her own sweet will, but for convalescent children of an inquiring turn of mind a better plan is that followed in the cot shown in the second drawing, where, by a simple but less obvious movement, the side pieces can be made to slide down below the level of the mattress. No amount of pressure from above will set it in motion, and the necessary manipulation may easily remain an unsolved mystery to the child. Our sketches are given by kind permission of Messrs. Heal, and Messrs. Shoolbred, both well-known and thoroughly reliable makers. It is, of course, most essential, in choosing cots for sick children, to see to it that all adjusting

Cot with Sliding Sides.

processes shall be capable of being carried out with absolute smoothness and absence of jarring. Hence a little more money laid out on a really well-made bedstead or cot is never ill-spent. It is impossible to expect that very great cheapness can be combined with satisfactory work and wear, although the comparative prices of the excellent bedsteads and cots to be seen both at Heal's and Shoolbred's are wonderfully moderate. No doubt, both for appearance and easy manipulation, brass has an advantage over plain iron; but the extra labour in keeping it as bright as it should be is a consideration, and in cases where the work is heavy, and wardmaids few, it may be better to keep to the simplest iron frame obtainable.

For the greater protection of very small children with restless propensities a frame has been devised to stretch over the top of the cot, filled in with soft netting, to effectually prevent any scrambling over the sides to the peril of life and limb. Being quite soft and stretchable it is a harmless restraint, and may be useful in cases where the nurse's attention cannot always be undivided. Another design we have seen for dropping the side of a cot provides a hinged support underneath the frame, which, on being pushed in, allows the side to drop down upon the floor. These various methods are all more or less good; they all make for the greater safety of the children, and the increased convenience of their attendants. Some are naturally better adapted for private use, others for the hospital ward, but the choice as to design is almost limitless, and every practical requirement would seem to be met in those we have specially commented upon above.

## Figures and Tables

**Figure f1:**
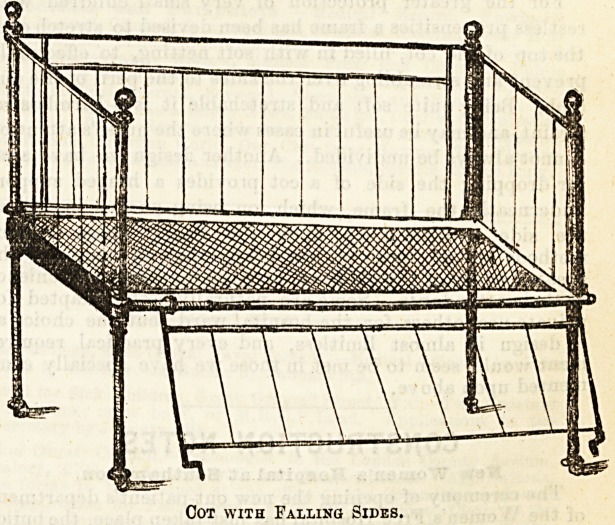


**Figure f2:**